# Fine and ultrafine particle exposure during commuting by subway in Vienna

**DOI:** 10.1007/s00508-019-1516-3

**Published:** 2019-06-07

**Authors:** Klaus-Peter Posselt, Manfred Neuberger, David Köhler

**Affiliations:** 1 Center for Public Health, Medical University of Vienna, Kinderspitalgasse 15, 1090 Vienna, Austria; 2 Felbigergasse 3/2/18, 1140 Vienna, Austria

**Keywords:** Air pollution, PM2.5, Nano-particles, Metro, Personal exposure

## Abstract

Mass concentrations of particulate matter (PM_10_, PM_2.5_, PM_1_), lung deposited surface area and particle number concentrations were measured for the first time in all Viennese subway lines inside cabins and in two subway stations, one aboveground and the other underground. The observed data were examined for significant differences between the exposure to fine particulate matter and ultrafine particles. Analysis of the trip averages in the five lines U1, U2, U3, U4 and U6 showed significant differences for PM_10_, PM_2.5_ and PM_1_ (all three mass concentrations: *p* < 0.001). Medians for PM_10_, PM_2.5_ and PM_1_ were highest in the U1 (73.6, 38.9, 27.1 µg/m^3^, respectively) and U3 (113.3, 47.1, 26.7 µg/m^3^, respectively) and significantly higher in the underground subway station than in the subway station on ground level. Regarding ultrafine particles no significant differences were found between the subway lines and no significant differences between the underground subway station and the subway station on ground level; however, new air-conditioned cabins had lower particle number concentrations and both particle number concentrations and lung deposited surface area were higher in cabins with open windows.

## Introduction

Air pollution is one of the main etiological factors in today’s worldwide mortality [1]. Long-term exposure [2, 3] and short-term exposure [4–7] to fine particulate matter (FPM) are associated with an increase in mortality. The FPM is usually measured as a mass concentration (MC) of all particles smaller than 10 µm (PM_10_) or 2.5 µm (PM_2.5_). In addition, the MC of all particles smaller than 1 µm (PM_1_) were also determined.

Although there is some evidence on negative health effects of ultrafine particles (UFP) [8], evidence for an increase in mortality from UFP is weaker than from FPM and to define the impact of UFP on health further studies are needed [6, 9]. There are also only few studies available on lung deposited surface area (LDSA), a newer unit to define the outer surface of particles influencing cells in the respiratory tract [10, 11]. In urban atmospheres and indoors the high variability of particle number and LDSA in space and time makes it difficult to quantify their impact on health. Not only the size of particles seems to play a role in the toxicity but also the sources, since FPM from mobile sources or coal combustion have a greater impact on mortality than FPM from crustal material [12].

In Vienna, exposure to FPM was found to be associated with an increase in acute and subacute mortality [13] and hospital admissions [14]. Strasser et al. already tested four different types of commuting in Vienna for FPM and UFP pollution. The results showed higher PM_2.5_ and PM_1_ in the subway when compared to buses. The PM_10_, PM_2.5_ and PM_1_ concentrations were higher in the subway when compared to cars. Also, particle number concentration (PNC) was higher in trams than in the subway and LDSA was higher on bicycles than when commuting by subway but this exploratory study covered only a small part of the Vienna subway system [15].

In Milan, a study found higher FPM concentrations in the subway in comparison to cars and open-air active modes whereas open-air active modes had the highest UFP levels compared to subway and car, indicating motor traffic influence on open-air active modes [16]. A study in Athens found 3–10 times significantly higher PM concentrations on the underground platforms of the subway system compared to outdoor measurements [17]. In Barcelona, similar results were detected [18], with PM_2.5_ samples collected in the subway system showing high Fe, Cu, Ba, Mn, Zn and Cr concentrations, possibly released by rail, wheels or brake pads, and the oxidative potential of the particles has been evaluated [19]. A study in Helsinki showed similar PNCs and size distributions at the underground subway station compared to the urban background monitoring site. The PM_2.5_ was higher at the underground stations compared to the ground level station and to subway wagons and the most enriched element in the samples was iron [20].

This was the first field study in the total Viennese subway system to examine for differences in personal exposure to PM_10_, PM_2.5_, PM_1_, PNC and LDSA.

## Material and methods

To evaluate potential risks to health, the 24-hour mean guidelines for PM_10_ (50 µg/m^3^) and PM_2.5_ (25 µg/m^3^) provided by the World Health Organization (WHO) were used [21] but it has to be considered that commuting time is shorter than 24 h. Since the effect of exposure can vary between individuals and since no indications for thresholds have been found for health effects of PM and UFP, the WHO declares that it is unlikely that any guidelines will lead to complete protection for every individual [21].

Subway line U5 is still under construction, unfinished and could not be included. Also excluded was the U6 concerning LDSA and PNC for comparing air-conditioned with not air-conditioned trains because the U6 uses a different type of train than the other subway lines. Regarding LDSA and PNC for comparing cabins with open windows with cabins with windows closed, only data from not air-conditioned trains were used.

### Collection of data

Data were obtained on consecutive workdays in August 2016 for PM_10_, PM_2.5_, PM_1_, PNC and LDSA in all subways and two stations. Measurements were carried out from start to end of the line at three trips per subway line and in two subway stations, one underground (Taborstraße, three stores below street level) and one on street level (Michelbeuern-AKH, open air rail with roofed platforms). Of the three measurements in the subways one took place in an opposing direction to the two other measurements. While measurements in the subway started at approximately 09:00, 12:00 and 15:00 and lasted until the end of the train ride (mean duration of subway rides = 30.6 min; details in Table 1), measurements in the subway stations lasted exactly 30 min starting at 11:00, 12:00 and 13:00 on 26 and 29 August 2016. In the stations 300 PM_x_ measurements and 1800 PNC and LDSA measurements were therefore performed per run. Measuring devices were placed in the most central subway cabin and near the middle of the stations on seating accommodation.

**Table 1:** Measurements in the subways U1 and U3 (mainly underground) U4 and U6 (more aboveground) and U2 (newest line, equivalent underground and aboveground sections)

Measurements in the subways—date	Time	Subway—air-conditioning (AC)—windows open (yes/no)	From	To	Number of measurements: particle mass vs. particle number concentration and lung deposited surface area
Measurement 1—19.08.2016	9:01:06–9:40:37	U6—AC—yes	Floridsdorf	Siebenhirten	389 vs. 2372
Measurement 2—19.08.2016	12:00:59–12:37:18	U6—AC—no	Floridsdorf	Siebenhirten	365 vs. 2190
Measurement 3—19.08.2016	15:13:32–15:52:31	U6—AC—yes	Siebenhirten	Floridsdorf	391 vs. 2424
Measurement 4—22.08.2016	9:06:37–9:34:18	U4—no AC—no	Heiligenstadt	Schönbrunn	270 vs. 1662
Measurement 5—22.08.2016	12:22:54–12:46:22	U4—AC—no	Schönbrunn	Heiligenstadt	237 vs. 1409
Measurement 6—22.08.2016	14:55:11–15:19:10	U4—AC—no	Heiligenstadt	Schönbrunn	241 vs. 1440
Measurement 7—23.08.2016	9:17:39–9:46:12	U3—no AC—yes	Simmering	Ottakring	287 vs. 1714
Measurement 8—23.08.2016	12:25:40–12:54:17	U3—no AC—yes	Ottakring	Simmering	288 vs. 1718
Measurement 9—23.08.2016	15:14:39–15:41:44	U3—no AC—yes	Simmering	Ottakring	270 vs. 1626
Measurement 10—24.08.2016	9:20:39–9:51:53	U2—AC—no	Seestadt	Karlsplatz	311 vs. 1875
Measurement 11—24.08.2016	12:05:40–12:36:29	U2—AC—no	Karlsplatz	Seestadt	310 vs. 1850
Measurement 12—24.08.2016	15:10:48–15:42:08	U2—AC—no	Seestadt	Karlsplatz	316 vs. 1881
Measurement 13—25.08.2016	9:20:29–9:54:08	U1—no AC—yes	Reumannplatz	Leopoldau	339 vs. 2020
Measurement 14—25.08.2016	12:08:17–12:35:35	U1—no AC—yes	Leopoldau	Reumannplatz	274 vs. 1639
Measurement 15—25.08.2016	15:09:18–15:38:09	U1—AC—no	Leopoldau	Reumannplatz	291 vs. 1732

While the U1 and U3 are mainly underground lines, the tracks of the U4 and U6 consist of more aboveground track sections. The U2 consists of almost equivalent underground and aboveground sections and is the newest line. Due to construction works it was not possible to perform measurements in both directions in the track sections (including stations) Oberlaa to Troststraße and Hütteldorf to Hietzing and two stations were passed through without a stop in one driving direction.

### Measuring devices

The PM_10_, PM_2.5_ and PM_1_ were measured using an optical particle counter, the GRIMM Aerosol Portable Laser Aerosolspectrometer and Dust Monitor®, Model 1.108 (GRIMM Aerosol Technik Ainring GmbH & Co. KG, Ainring, Germany). It detects particles with aerodynamic diameters over 300 nm. The performance had already been evaluated and compared to another model [22]. The mass concentrations were determined using 6 s intervals. The PNC and LDSA were determined using the miniDiSC® (Dr. Martin Fierz, Fachhochschule Nordwestschweiz, Windisch, Switzerland), measuring in 1 s intervals. The miniDiSC is a diffusion size classifier able to measure number concentrations of UFP between 10 nm and 300 nm as well as LDSA.

### Statistics

Data were checked and one extremely high PM_10_ value was defined as an outlier and removed. For PM_10_, PM_2.5_ and PM_1_, medians for each subway line and station were determined. The data were tested for Gaussian distribution with Kolmogorow-Smirnow tests and tested for significant differences using Mood’s median test. Afterwards, post-hoc analyses were performed on the subway data using the Bonferroni correction to determine pairwise significant differences between the lines. For PNC and LDSA, Gaussian distribution was also tested using Kolmogorow-Smirnow tests and the one-way analysis of variance was used to test for significant differences between the lines and performed Scheffé post hoc analyses afterwards. For comparing the stations, closed with opened windows and for comparing not air-conditioned with air-conditioned trains t‑tests were used.

## Results

**Fig. 1:**
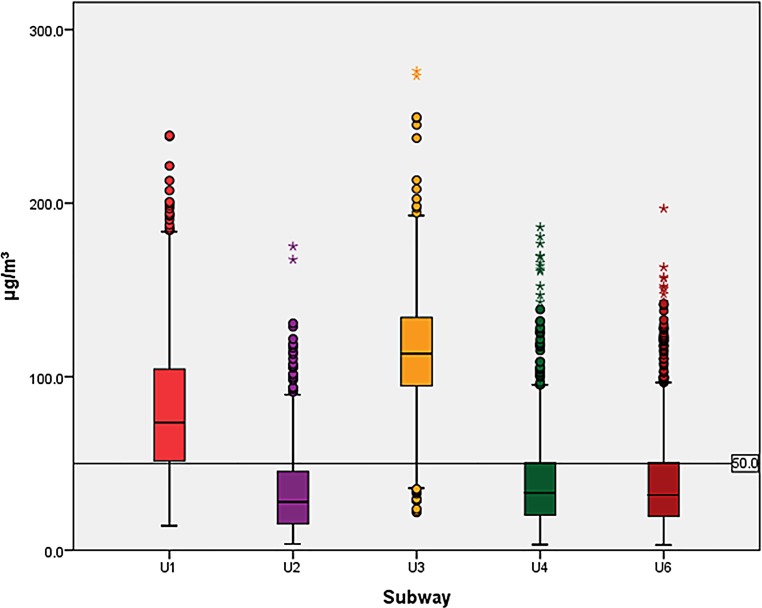
Particle mass below 10 µm aerodynamic diameter (PM_10_), boxplots for all subway lines related to the PM_10_ 24-h mean threshold [21]

**Fig. 2:**
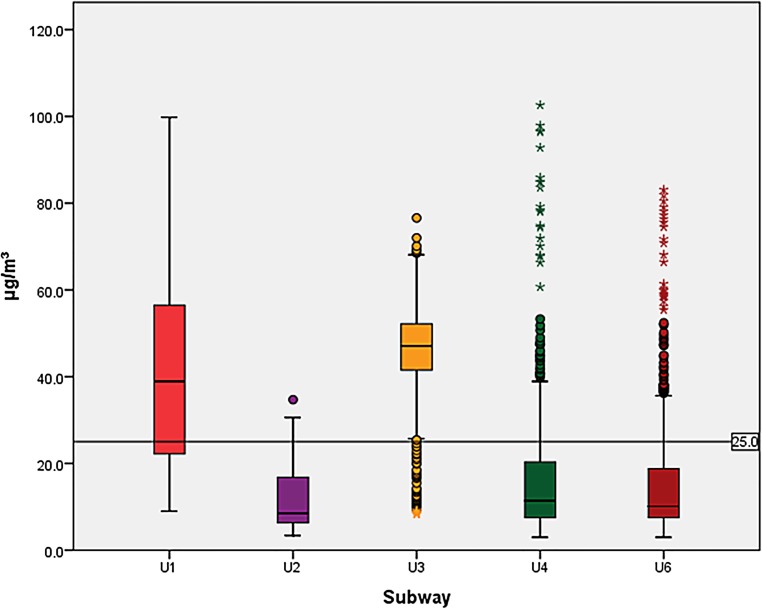
Particle mass below 2.5 µm aerodynamic diameter (PM_2.5_), boxplots for all subway lines related to the PM_2.5_ 24-h mean threshold [21]

**Fig. 3:**
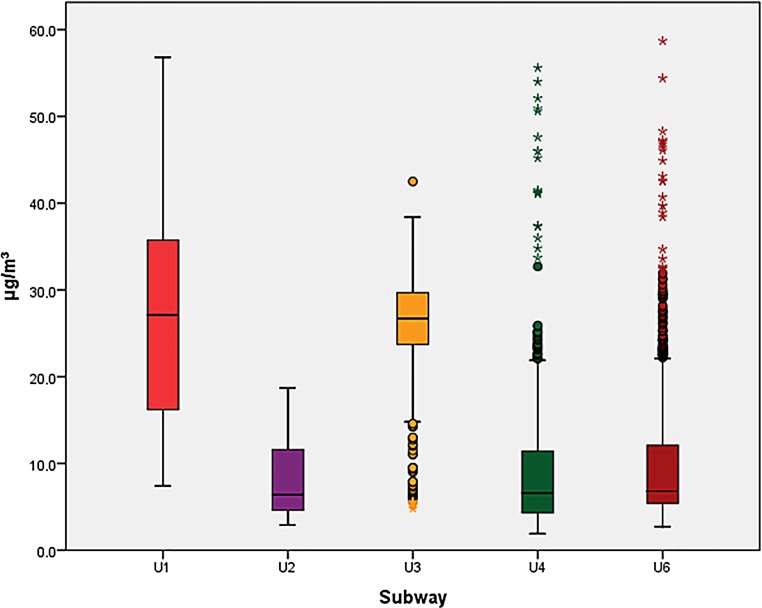
Particle mass below 1 µm aerodynamic diameter (PM_1_), boxplots for all subway lines

Except for PM data on 6 subway rides, which were 3–48 s too short due to technical problems, data collection was complete. The comparison of FPM in the subways showed significant differences between all subways (for all three MC: *p* < 0.001). Post hoc analysis of subways in pairs showed significant differences between the medians of all subways for PM_10_ except the lines U6 and U4. Also, for PM_2.5_ significant differences were found for all pairs except U2 and U6. For PM_1_, more inhomogeneous results were obtained where the pairs U2 and U3, U2 and U1, U4 and U3, U4 and U1, U6 and U3 and U6 and U1 showed significant differences. The PM_10_ and PM_2.5_ in the U1 and U3 particularly exceeded the WHO guidelines; however, it must be mentioned that the WHO guidelines apply to a 24-h time period while each subway measurement lasted only half an hour. For PM_1,_ the medians in the U1 and U3 even surpassed the PM_2.5_ guidelines. The FPM exposure is shown in Figs. 1, 2 and 3.

Furthermore, the analysis of the stations showed significant differences (all three MC: *p* < 0.001). All three MC medians were approximately three times higher in the underground station than in the ground level subway station (underground subway station vs. ground level subway station: PM_10_: 61.5 µg/m^3^ vs. 20.2 µg/m^3^, PM_2.5_: 24.6 µg/m^3^ vs. 7.9 µg/m^3^, PM_1_: 14.2 µg/m^3^ vs. 5.0 µg/m^3^).

Near the underground subway station considerably lower concentrations were measured simultaneously at ground level by the ambient air monitoring network of the municipality (underground subway station vs. ambient air monitoring network: PM_10_: 61.5 µg/m^3^ vs. 17.2 µg/m^3^, PM_2.5_: 24.6 µg/m^3^ vs. 6.5 µg/m^3^). Near the ground level subway station, the monitoring network of the municipality registered similar PM_2.5_ (ground level subway station vs. monitoring network: 7.9 µg/m^3^ vs. 8.7 µg/m^3^) as were measured inside the station but lower PM_10_ (ground level subway station vs. monitoring network: 20.2 µg/m^3^ vs. 11.7 µg/m^3^).

**Fig. 4:**
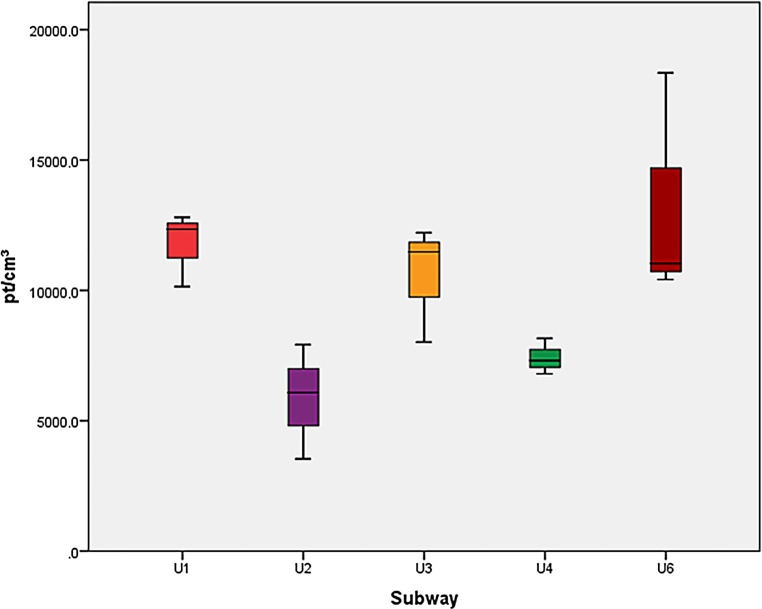
Particle number concentration (PNC), boxplots for all subway lines

**Fig. 5:**
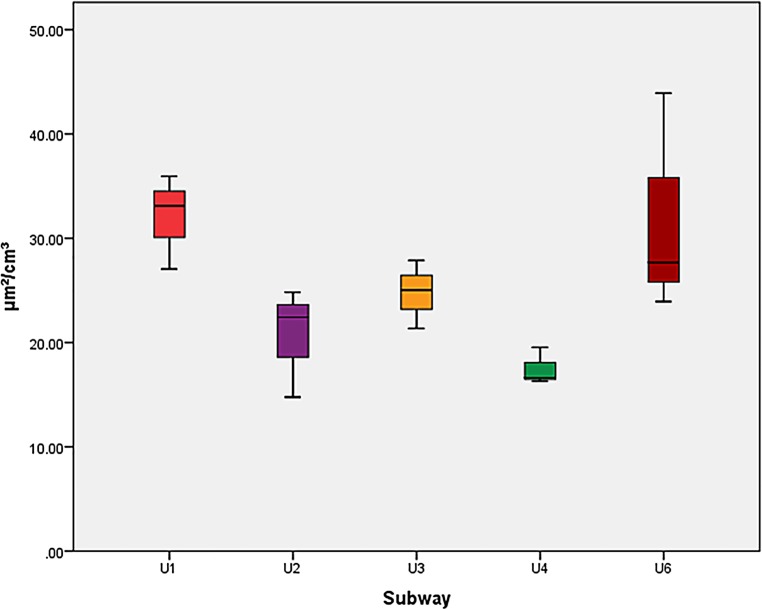
Lung deposited surface area (LDSA), boxplots for all subway lines

Regarding UFP, no significant differences were found between the lines in the post hoc tests and no significant differences between the underground and ground level subway station. The PNC and LDSA means and the standard deviations in subway stations are listed in Table 2. A confidence interval of 95% was used. The LDSA and PNC exposures are shown in Figs. 4 and 5.

**Table 2:** Particle number concentration (PNC) and lung deposited surface area (LDSA), mean values in two subway stations

Subway station	PNC (pt/cm^3^)	PNC standard deviation (pt/cm^3^)	LDSA (μm^2^/cm^3^)	LDSA standard deviation (μm^2^/cm^3^)
Taborstraße	10,000	2667	23.3	3.3
Michelbeuern-AKH	8309	1870	23.5	5.4

*PNC* particle number concentration

Significant differences between air-conditioned and not air-conditioned trains (*p* = 0.023) were found regarding PNC but no significant differences regarding LDSA. Significant differences were also found regarding LDSA (*p* = 0.02) and PNC (*p* = 0.005) exposure between cabins with open windows and cabins with closed windows.

## Discussion

The highest medians for PM_10_, PM_2.5_ and PM_1_ were found in the U1 and U3 subways. This may be because of the long consecutive tunnel sections on the routes of the U1 and U3. The lack of cleaning or the insufficient air conditioning in the tunnels may lead to a deposition of FPM. The wind of following trains may lead to resuspension of the particles that will then enter the train cabins and therefore the tidal air of passengers. This study found similar results as in Athens [17], Barcelona [18] and Helsinki [20] when comparing the underground station to the ground level station showing higher FPM levels in the underground station. Studies have also shown that air conditioning in trains can reduce FPM levels and could therefore be a potential air cleaning method in subways [17, 18]. This is supported by these results of lower FPM on lines with air-conditioned trains and of lower PNC and LDSA in cabins with closed windows.

The chemical composition of FPM inside the subway system of Vienna still needs to be examined. Some studies showed that the main metal element present in FPM found in subway systems was iron [20, 23, 24]. This could be due to the abrasion of rail tracks, wheels and braking pads inside the subway system [18].

The data collection took place in summer only, so further studies during all seasons should be performed. Equipping stations and trains with measurement devices could be an appropriate way to gather data to more precisely determine air quality in the subway system. Additionally, chemical analysis of the particles found in the subway system should be performed to determine and reduce potential health risks caused by FPM and UFP.

Finally, two benefits of commuting by subway have to be mentioned: Shorter travelling times compared to bus and car reduce the cumulative dose for passengers during commuting and the emission of air pollutants by a subway driven by electricity from water power is much lower than by individual traffic with cars, mainly driven by diesel or gasoline [15, 16]. Since the scientific evidence for UFP’s precise effects on health is incomplete and since there are as yet no ambient air standards for either PNC or LDSA, the data in this study need to be reanalyzed and the potential health effects re-evaluated when more exact scientific evidence is revealed.
